# Navigation of the Clinical Implications, Interventional Challenges, and Complexities of the Circumflex Coronary Artery: A Comprehensive Review

**DOI:** 10.31083/RCM47426

**Published:** 2026-02-11

**Authors:** Abdelrahman Elhakim, Ahmad Hassaan, Ibrahim Yassen, Mohamed Mosaad, Mohamed Elhakim, Osama Bisht, Mahmoud Baraka, Mohammed Saad

**Affiliations:** ^1^Cardiology Department, Staedtisches Klinikum Braunschweig, 38126 Braunschweig, Germany; ^2^Cardiology Department, Al-Azhar University, Nasr City, 11884 Cairo, Egypt; ^3^Intensive Care Medicine Department, The Royal Prince Alfred Hospital, Sydney, NSW 2050, Australia; ^4^Cardiology Department, Coswig Heart Center, 06869 Coswig, Germany; ^5^Cardiology Department, Ain Shams University, Abbaseya, 11517 Cairo, Egypt; ^6^Cardiology Department, Schleswig-Holstein University Hospital-Kiel, 24105 Kiel, Germany

**Keywords:** great cardiac vein, circumflex artery, calcific lesion, pericardial effusion, mitral valve, left atrial appendage, chronic total occlusion, intravascular ultrasound, percutaneous coronary intervention, transthoracic echocardiography, computed tomography

## Abstract

The circumflex (Cx) coronary artery is more vulnerable to injury than other coronary arteries during procedures such as radiofrequency ablation, left atrial appendage closure, mitral valve repair, and coronary sinus-based mitral valve intervention. Furthermore, a lower success rate was also observed in the Cx artery during chronic occlusion recanalization. Additionally, injury to the great cardiac vein during Cx artery interventions can occur due to the highly variable and often unpredictable relationship between the great cardiac vein and the Cx artery, which occurs in approximately 30% of cases. Imaging information on the Cx artery and the associated relationship with surrounding cardiac structures is crucial for understanding spatial orientation. This knowledge aids preventive measures, accurate prediction, prompt recognition, and understanding of injury mechanisms, thereby facilitating appropriate therapeutic interventions. We present a comprehensive literature review of the clinical implications, complexities, and challenges associated with the Cx artery, which could help in management strategies and improve outcomes.

## 1. Introduction

In most individuals, the left main coronary artery bifurcates into two major coronary arteries: the left anterior descending artery (LAD) and the circumflex artery (Cx). The circumflex artery can be referred to using multiple terms, including the Cx, ramus circumflex artery (RCx), and left circumflex artery (LCx), and is often colloquially called the “circ” [1].

This artery traverses the left atrioventricular groove between the left ventricle and left atrium in the epicardium. It gives off up to three obtuse marginal branches, and if it has coronary dominance with the right coronary artery, it may give off a left posterolateral branche (PLB) and supply the posterior descending artery. It supplies the lateral and posterolateral walls of the left ventricle [1].

Anatomical variations and anomalies in the Cx coronary artery can be challenges in management strategies, such as missing of anomalous Cx ischemia, separate ostia, tortuous arteries, variations in anatomical course, calcium burden, bifurcation, and angulation.

In addition, the relationship between the great cardiac vein and the circumflex artery is highly variable and unpredictable in 30% of cases [2]. Thus, injury to the great cardiac vein during circumflex coronary artery intervention could be caused by a severe calcific Cx lesion that protrudes outside the arterial wall during the intervention. This causal relationship has not been adequately discussed in the literature.

Moreover, in the literature, the Cx coronary artery is more likely to be injured during radiofrequency ablation [3], left atrial appendage closure [4], mitral valve repair [5, 6], and coronary sinus-based mitral valve interventions [7], and it has a lower success rate during chronic total occlusion recanalization [8].

Therefore, imaging information about the Cx artery and its relation to surrounding cardiac structures is useful to obtain a better understanding of its spatial orientation and to help in the management of heart diseases with dedicated de-bulking devices and structural heart interventions.

**Fig. 1.**
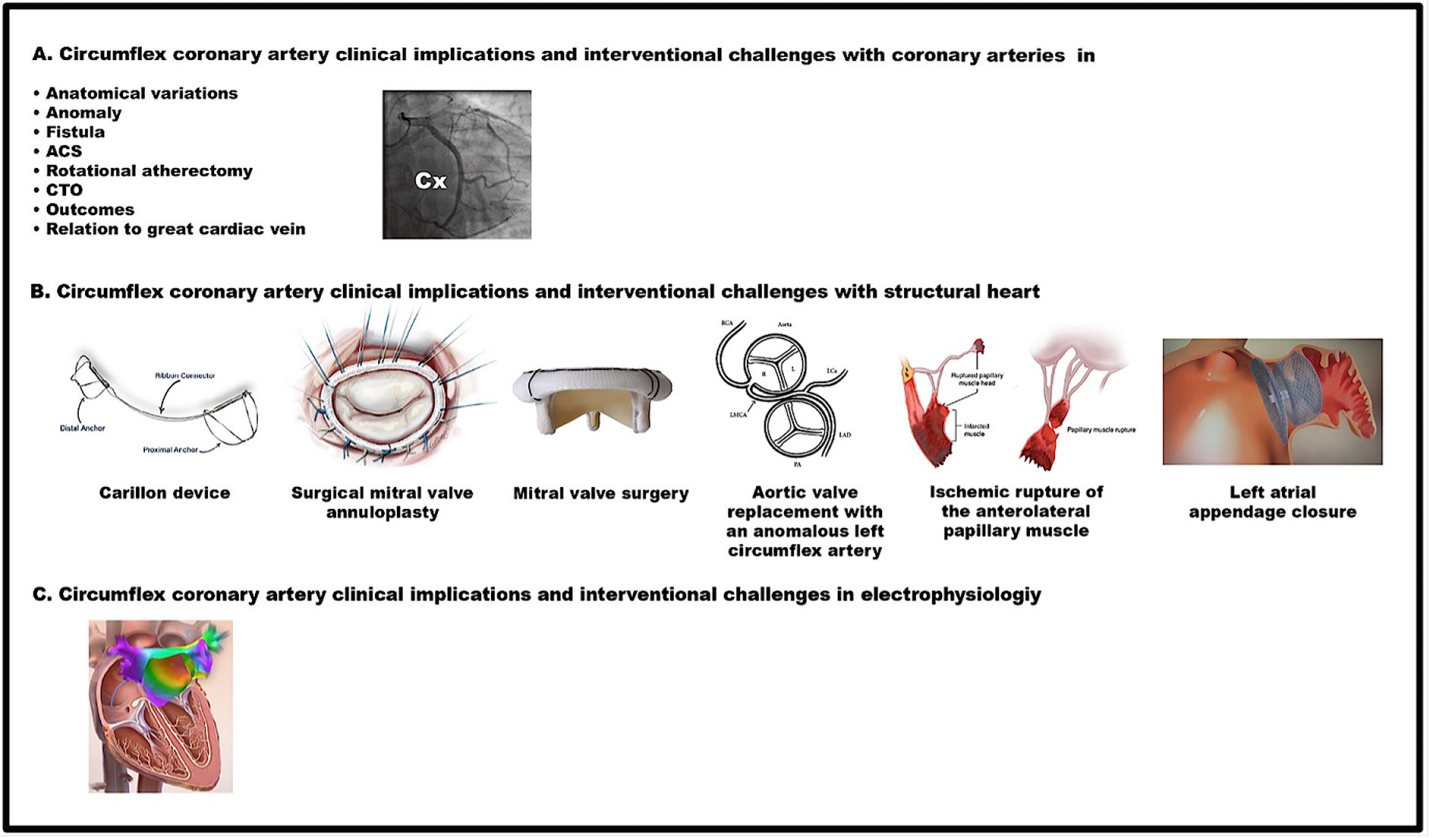
**General illustration of circumflex coronary artery peculiarities**. (A) coronary arteries; (B) structural heart interventions, and (C) electrophysiological radio-frequency ablation. ACS, acute coronary syndrome; CTO, chronic total occlusion.

Highlighting the Cx peculiarities (Fig. 1) and taking imaging information into consideration could improve management strategies by facilitating preventive measures, prediction, prompt recognition, and understanding of the injury mechanism, if applicable. The implementation of dedicated therapy according to the cause could improve outcomes.

In this review, we summarize the main Cx peculiarities, which are divided into three main parts: (A) coronary artery disease, (B) structural heart disease, and (C) electrophysiology. Table 1 summarizes the clinical implications and management strategies.

**Table 1.** **Summarizes the clinical implications, interventional challenges, and management strategies for the Cx coronary artery**.

Category		Management
A. Clinical implications of the Cx coronary artery		
	Determines codominance	cCT and CA
	Anomalous origin	cCT and CA
	Anomalous double Cx	
	Anomalous aortic origin of a coronary artery (AAOCA)	
	Anomalous left coronary artery from the pulmonary artery	cCT, right heart catheter and CA
	Fistula draining into coronary sinus, right atrium, pulmonary artery and/or left atrial appendage	cCT, right heart catheter and CA
	Acute coronary syndrome and Cx occlusion	ECG, especially posterior leads (V7–V9), TTE, cardiac biomarkers and CA
	Culprit lesion Cx or RCA?	ECG, TTE, noninvasive ischemic test (PET, SPECT, cCT or cMRI), intracoronary imaging (IVUS or OCT) and or physiological flow assessment (FFR/IFR).
	Rotational atherectomy PCI outcomes	cCT, intracoronary imaging and selection of Debulking strategy
	Cx and chronic total occlusion	
	Great cardiac vein injury	TTE, cCT, intracoronary imaging and selection of Debulking strategy
B. Cx and structural heart disease		
	Mitral valve annuloplasty	cCT, non-resection techniques, minimally invasive strategies that avoid annular manipulation such as transapical artificial chordae implantation.
	Mitral valve surgery	ECG changes and intraoperative CA could help in the diagnosis, rescue PCI and emergency coronary bypass grafting
		In specific circumstances, repositioning the prosthesis or removing the annuloplasty ring could be imperative
	Indirect mitral annuloplasty using the Carillon device	ECG changes and intraoperative CA could help in the diagnosis.
		Treatment options include repositioning Carillon device, using another treatment strategy such as transcatheter edge-to-edge repair (Mitral Clip), Cx-Stenting or conservative therapy if the patient asymptomatic
	Ischemic rupture of the anterolateral papillary muscle	ECG, TTE, TEE, cMRI and CA
	Surgical aortic valve replacement (SAVR) with an anomalous Cx	cCT, CA, root aortography and usage of a smaller prosthesis
	Left atrial appendage closure	CCT intraoperative ECG changes, CA, repositioning of the device or using another device closure mechanism
C. Cx and Electrophysiology		
		cMRI, selection of radiofrequency ablation strategy, intraoperative ECG changes and CA, intraoperative spasmolytic medication, another ablation strategy and or Cx stenting

cCT, cardiac computed tomography; CA, coronary angiography; ECG, electrocardiogram; TTE, transthoracic echocardiography; TEE, transesophageal echocardiography; PET, Positron emission tomography; SPECT, single photon emission computed tomography; cMRI, cardiac Magnetic Resonance Imaging; IVUS, Intravascular ultrasound; OCT, Optical Coherence Tomography; FFR, Fractional flow reserve; IFR, The instantaneous wave-free ratio; PCI, percutaneous coronary intervention; Cx, circumflex artery; RCA, right coronary artery.

However, congenital Cx anomalies and their relevance in clinical practice are not comprehensively discussed in this review. For a more complete overview of the literature, we refer readers to the literature on congenital heart disease.

## 2. Clinical Implications, Interventional Challenges, and Complications of the Circumflex Coronary Artery 

### 2.1 Coronary Artery Disease and the Circumflex

#### 2.1.1 Anatomical Considerations: Cx Determines the Dominance of the Coronary Arteries

The origin of the supply to the posterior descending artery (PDA) and PLBs determines whether the coronary tree is right-dominant, left-dominant, or codominant [9].

Most people have a right-dominant coronary artery system, where the PDA is supplied by the right coronary artery (RCA). However, approximately 9% of the normal population is left-dominant (where the Cx provides the PDA) or co-dominant (where the PDA and/or posterolateral branch(es) supply is shared by the RCA and Cx [10].

#### 2.1.2 An Anomalous Origin of the Cx From the Right Sinus 

The most common coronary artery anomaly is the absence of the left main coronary artery, with the anterior and circumflex arteries originating separately in the left coronary sinus. The second most common anomaly is the circumflex artery arising from the right sinus of Valsalva (RSV). It is not a true anomaly but an anatomical variation of the coronary artery tree. This anomaly is more likely to result in earlier and more aggressive atherosclerosis than in normal coronary arteries due to a slit-like orifice and repeated compression of the retroaortic segment of the vessel leading to myocardial infarction or sudden cardiac death [11, 12].

Therefore, failure to predict and identify aberrant Cx during coronary angiography (CA) may hamper correct diagnosis (myocardial infarction with non-obstructive coronary arteries), delay intervention, increase infarct size and result in increased contrast volume and radiation exposure. In addition, the presence of an aberrant Cx from the RSV is challenging in transcatheter aortic valve implantation (TAVI). It should be recognized before the procedure to decrease the risk of coronary obstruction, take measures for coronary protection and proper selection of the transcatheter heart valve [13].

Triantafyllis *et al*. [13] analyzed the angiographic predictors of aberrant Cx and compared 136 patients with aberrant Cx and 135 controls. He suggested that a long LM-length and an acute bifurcation angle can indicate the presence of aberrant Cx. In addition, “Triantafyllis algorithm” has been proposed for the rapid identification of an aberrant Cx from the RSV. If the LM-length measured in a cranial view is >17.7 mm the patient carries a 5.3 times greater probability of having an aberrant Cx. However, if LM-length is <17.7 mm, a second measurement in a caudal view is necessary. In the caudal view, a LM-length >13.9 mm and an acute bifurcation angle ≤62.9° suggest an increased suspicion of an aberrant Cx. The absence of both indicates a low likelihood of an aberrant Cx [12].

#### 2.1.3 An Anomalous Double Cx

The occurrence of double or twin Cx coronary arteries is rare and is should be considered as an anatomical variation of the coronary artery tree. However, its clinical challenge is the chance of missing or underdiagnosis of anomalous Cx ischemia [12].

In general, one Cx artery originated from the left main artery, while the other artery originated from the RCA. In extremely rare cases, the two Cx arteries may originate from the left coronary sinus.

#### 2.1.4 An Anomalous Aortic Origin of A Coronary Artery (AAOCA)

In an AAOCA anomaly, the Cx originates from the right coronary sinus. The presence of systolic milking of the Cx during coronary angiography is a life-threatening condition, facilitates malignant arrhythmias and myocardial infarction, and should indicate an anomalous retro-aortic course [13].

#### 2.1.5 Anomalous Origin of the Left Coronary Artery From the Pulmonary Artery (ALCAPA)

The challenge in treating coronary artery anomalies is to identify them and determine their clinical relevance so that appropriate treatments can be administered.

An anomalous origin of the left circumflex coronary artery from the right pulmonary artery is an extremely rare coronary anomaly. The first presentation can be sudden cardiac arrest during life. This condition can be confirmed via multimodal imaging, and surgical correction is imperative [14, 15, 16]. Cardiac computed tomography (cCT), right heart catheter, and CA imaging play crucial roles in the diagnosis.

#### 2.1.6 Cx Fistula Draining Into the Coronary Sinus, Right Atrium, Pulmonary Artery, or Left Atrial Appendage

Coronary artery fistulas have different mechanisms of clinical manifestation, such as mural thrombosis at sites of coronary ectasia, rupture (aneurysmal wall degeneration), endocarditis, left-to-right shunting, myocardial ischemia secondary to coronary steal or side-branch obstruction (acquired), and aortic valve disruption (secondary to an aneurysmal proximal coronary artery) with insufficiency. cCT, right heart catheter, CA and intravascular ultrasound (IVUS) play a crucial role to evaluate, vessel size, mural clots, intimal integrity, and localized aneurysms. The nuclear stress test is, In contrast, typically negative for reversible ischemia.

Fistula closure is indicated if the pulmonary–systemic flow ratio (Qp:Qs) exceeds 1.5:1. In addition, aneurysmal degeneration can lead to mural thrombosis, rupture, and the steal phenomenon, which can lead to ischaemia and side-branch obstruction and necessitate intervention.

There are several case reports regarding Cx fistulas. Egorova *et al*. [17] reported a case of a large left Cx fistula draining into the coronary sinus with a hemodynamically significant intracardiac shunt that was treated with a ventricular septal defect occlude. Spapen *et al*. [18] reported a case of a giant left Cx aneurysm with a fistula in the right atrium. Hamadanchi *et al*. [19] reported a case of a congenital fistula that originated from the left Cx and drained into the left atrial appendage. Sigusch *et al*. [20] reported a case of a 60-year-old man presented with dyspnoea. Computed tomography (CT) and coronary angiography revealed the agenesis of the left pulmonary artery and a large fistula arising from the Cx artery, thereby ensuring left lung tissue supply.

For further reports of congenital Cx anomalies and their relevance in clinical practice, we refer readers to the congenital heart disease literature.

#### 2.1.7 Acute Coronary Syndrome (ACS) and Cx Occlusion

Acute Cx occlusions pose management challenges. If undiagnosed, they may lead to missing or delayed reperfusion. A standard 12-lead electrocardiogram (ECG) is less sensitive for infarctions involving acute Cx occlusions and can detect acute Cx occlusion in only one-third to one-half of ACS patients. Komatsu *et al*. [21] demonstrated that one-third of ACS patients with Cx occlusions showed no significant ST segment changes, resulting in delayed door-to-balloon time. In addition, patients with Cx occlusions tend to present with non-ST-elevation ACS (NSTE-ACS) compared with those with occlusions in other coronary arteries [19, 20, 22].

Bedside echocardiography to identify regional left ventricular asynergy and additional recordings of posterior leads (V7–V9) have been proposed to overcome these diagnostic challenges [22]. Additional echocardiographic regional wall motion abnormalities and cardiac biomarkers may help in the diagnosis.

#### 2.1.8 Culprit Lesions in Acute and Chronic Coronary Syndrome: Cx or RCA? 

Both the RCA and Cx are potential culprit lesions in patients with inferior myocardial infarction (MI). Coronary angiography alone may not be sufficient to determine the site of the culprit lesion. ECG algorithms have been proposed for identifying infarct-related arteries in patients with inferior MI. However, the discriminative power of these algorithms in identifying the actual culprit arteries in these patients remains unknown [23]. Percutaneous coronary intervention (PCI) guidance using noninvasive imaging such as cMRI, cCT, and positron emission tomography (PET), and intravascular imaging, such as IVUS and optical coherence tomography (OCT), as well as regional wall motion abnormalities in echocardiography, resulted in a reduction in the primary composite outcome of target lesion failure of 31% compared with angiography-guided PCI.

The advantage of quantitative imaging over conventional diagnostic imaging is the additional acquisition of biophysical parameters. This leads to a more objective diagnosis; helps in identifying stable vs. nonstable plaques, ulcers, dissections and thrombi and allows an assessment of the course of therapy.

However, the complexity and variety of quantitative imaging modalities necessitate familiarity and experience with these tools based on specific clinical settings, individual patient characteristics, and the availability of each procedure [21].

#### 2.1.9 Rotational Atherectomy and Cx

Rotational atherectomy (RA) in ostial Cx lesions in severe calcified ostial Cx lesions with substantial bending is challenging, necessitates operator experience, and plaque modification has higher complication risk compared to other coronary arteries. CCT and intracoronary imaging can help identify the anatomy, understand the underlying pathology, select the debulking strategy and appropriate burr size. In angiography, it is paramount important to have multiple projections to evaluate the actual contact point between the rota wire and calcification. If a severe eccentric calcification plaque is observed in the lateral wall of the Cx ostium, there is a risk of perforation on the carina side of the Cx ostium due to the jumping of the burr. Therefore, an attempt should be considered to ablate the lateral wall of the Cx ostium to avoid carina perforation. However, excessive lateral wall ablation can cause lateral wall perforation due to deep cuts. In addition, percutaneous bailout intervention of the Cx ostial perforation is challenging because the bending angle can interfere with stent delivery and an implantation of covered stents can occlude the LAD [24, 25].

#### 2.1.10 PCI Outcome of Cx

Percutaneous interventions for ostial coronary artery lesions are challenging because of the elastic fiber content of the ostium, calcium burden, bifurcation, and angulation.

A study of 4759 patients with 1-year follow-up for procedural outcomes for de novo ostial or very proximal Cx lesions demonstrated higher rates of target lesion revascularization and major adverse cardiac events compared with ostial or very proximal LAD or RCA lesions [26]. The potential causes could be management challenges in acute Cx occlusion, and when this condition remains undiagnosed, it may lead to missing or delayed reperfusion. Quantitative imaging can help obtain a more objective diagnosis and improve management outcomes.

#### 2.1.11 Chronic Total Occlusion (CTO) Outcome and Cx

In the multicenter CTO-PCI registry, the Cx was the least common target vessel compared with the LAD and RCA. In addition, PCI of Cx-CTOs was associated with a lower procedural success rate and a nonsignificant trend of higher rates of complications [8]. This may have been because the Cx lesions were more tortuous and exhibited variation in anatomical course, calcium burden, bifurcation, and angulation. cCT imaging using 3D volume rendering images could help in procedure planning and management. It provides a detailed anatomical and morphological characterisation of the plaque morphology and content. An application of scoring systems can help in selection of debulking strategy and to predict the likely success of the intervention.

#### 2.1.12 Great Cardiac Vein and Cx

**Fig. 2.**
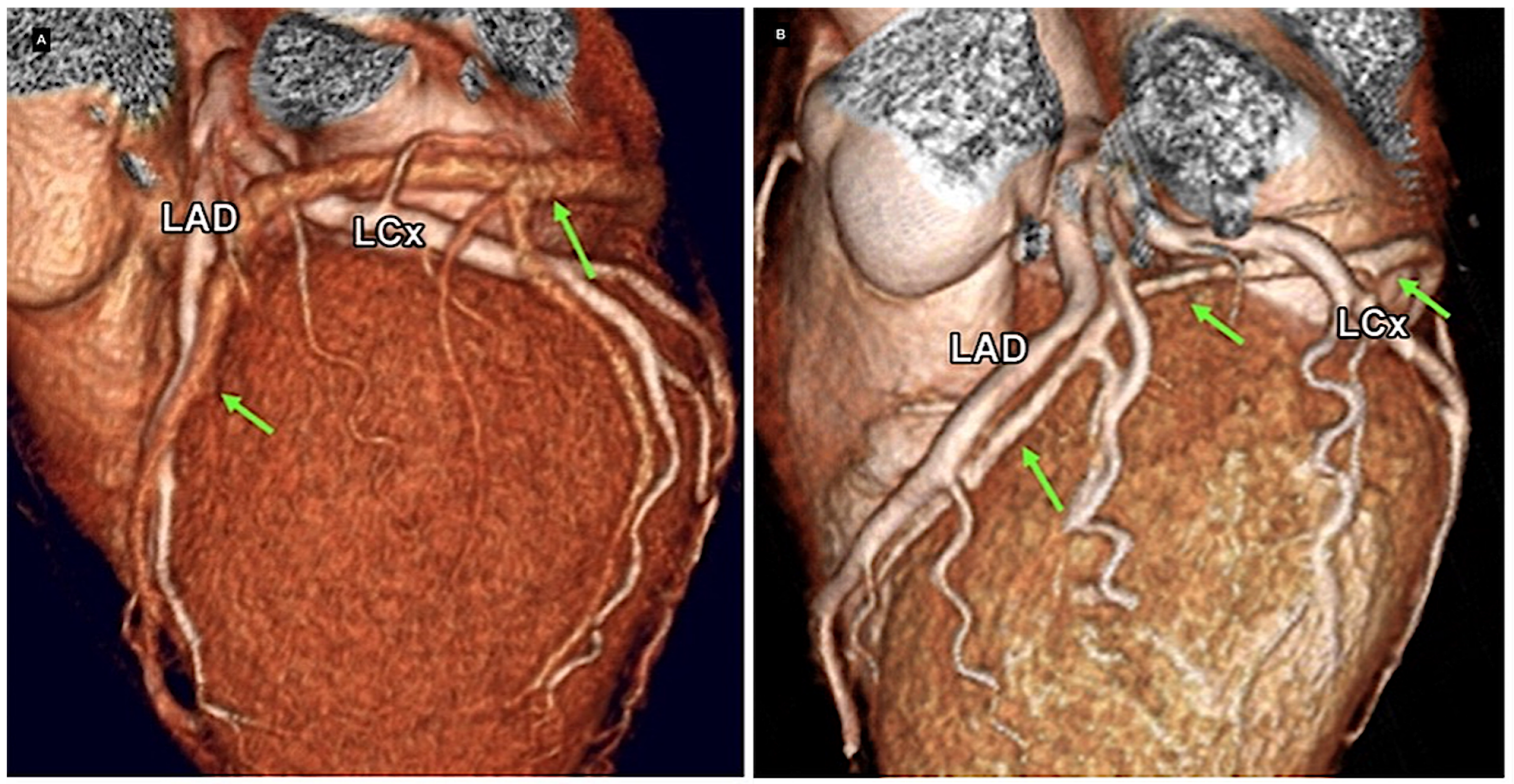
**Computed tomography (CT) mapping of the coronary veins**. The great cardiac vein (GCV) (green arrows) crossed the left anterior descending (LAD) and left circumflex (LCx) arteries to form a triangle. The anterior interventricular vein courses superficially to the arteries in 60%–70% of the population (A) and passes under both arteries in 30% of the population (B) [2].

The great cardiac vein (GCV) is one of the longest coronary sinus tributaries and mostly originates in the lower third of the anterior interventricular sulcus (58%). The great and middle cardiac veins merge at the base of the heart, forming the coronary sinus. The GCV crosses the LAD and the Cx branches of the left coronary artery, forming the triangle of Brocq [27]. The relationships between the vein and arteries are highly variable and are practically unpredictable in 30% of population [2]. The GCV lies superficial to the arteries in 60%–70% of the population (Fig. 2A, Ref. [2]) and passes under both arteries in 30% of the population (Fig. 2B). This is a peculiarity of the Cx coronary artery compared with other coronary arteries [2].

These close relationships can cause injury to the GCV during PCI in severely calcified circumflex arteries as follows: both Cx and GCV pass through the anterior interventricular sulcus. While performing aggressive balloon dilatation, this could make the GCV more vulnerable to injury as the sulcus is a narrow space and both vein and artery have a direct contact without enough space to move. The calcific plaque could protrude outside the artery and could injured the neighboring GCV.

Imaging information about the cardiac venous system (CVS) could help in management strategies understand the spatial orientation, and calcium last to minimize complications and improve outcomes [28].

Injury of GCV during Cx-PCI is a diagnosis of exclusion if a venous pericardial effusion occurred without injury to the right side of the heart or the surrounding structures, and a thoracic CT demonstrates a hematoma in the Cx-PCI region. A hematoma can deteriorate the hemodynamic status without effusion (dry tamponade). Management strategy is a case-by-case decision. First, consider conservative therapy and consider pericardial drainage If the patient bleeds progressively or is hemodynamically unstable. Further exploratory pericardiotomy may be imperative in difficult scenarios to evacuate the hematoma and seal the injured vein. Other catheter-based interventions could be helpful bailout techniques in high-risk patients [28].

### 2.2 Structural Heart Disease and Cx

#### 2.2.1 Surgical Mitral Valve Annuloplasty and the Anomalous Aortic Origin of Cx

Mitral valve annuloplasty can result in iatrogenic injury to the Cx in certain populations. In patients with an anomalous aortic origin of a coronary artery and those undergoing mitral valve annuloplasty due to high-grade symptomatic mitral valve regurgitation, mitral valve annuloplasty can result in iatrogenic injury to the Cx because it lies within the atrioventricular groove. The risk of mechanical Cx occlusion seems to be higher in patients with a Cx with an anomalous course. Although predictable, this complication is poorly described in the literature, and its incidence is unknown [13]. CCT imaging before surgery can help in the management strategy.

For example, in such anomalies, mitral valve repair is challenging, and a partial annuloplasty ring or band should be considered. Non-resection techniques should be considered if repair durability is not compromised. Minimally invasive strategies that avoid annular manipulation, such as transapical artificial chordae implantation, may be reasonable alternatives.

#### 2.2.2 Surgical Mitral Valve Surgery and Left-Dominant Cx 

Iatrogenic left Cx injury after mitral valve repair is associated with blind annuloplasty suture ligation or kinking of the Cx, and it is more common in the left-dominant coronary artery circulation. The clinical presentation of this condition can include early ST segment changes, malignant ventricular arrhythmias, and segmental wall motion abnormalities [5]. ECG changes and intraoperative CA may help in the diagnosis.

Possible treatment options for this life-threatening complication include rescue PCI and emergency coronary bypass grafting. In specific circumstances, repositioning the prosthesis or removing the annuloplasty ring may be imperative [6].

#### 2.2.3 Transcatheter Indirect Mitral Annuloplasty Using the Carillon Device and Cx

**Fig. 3.**
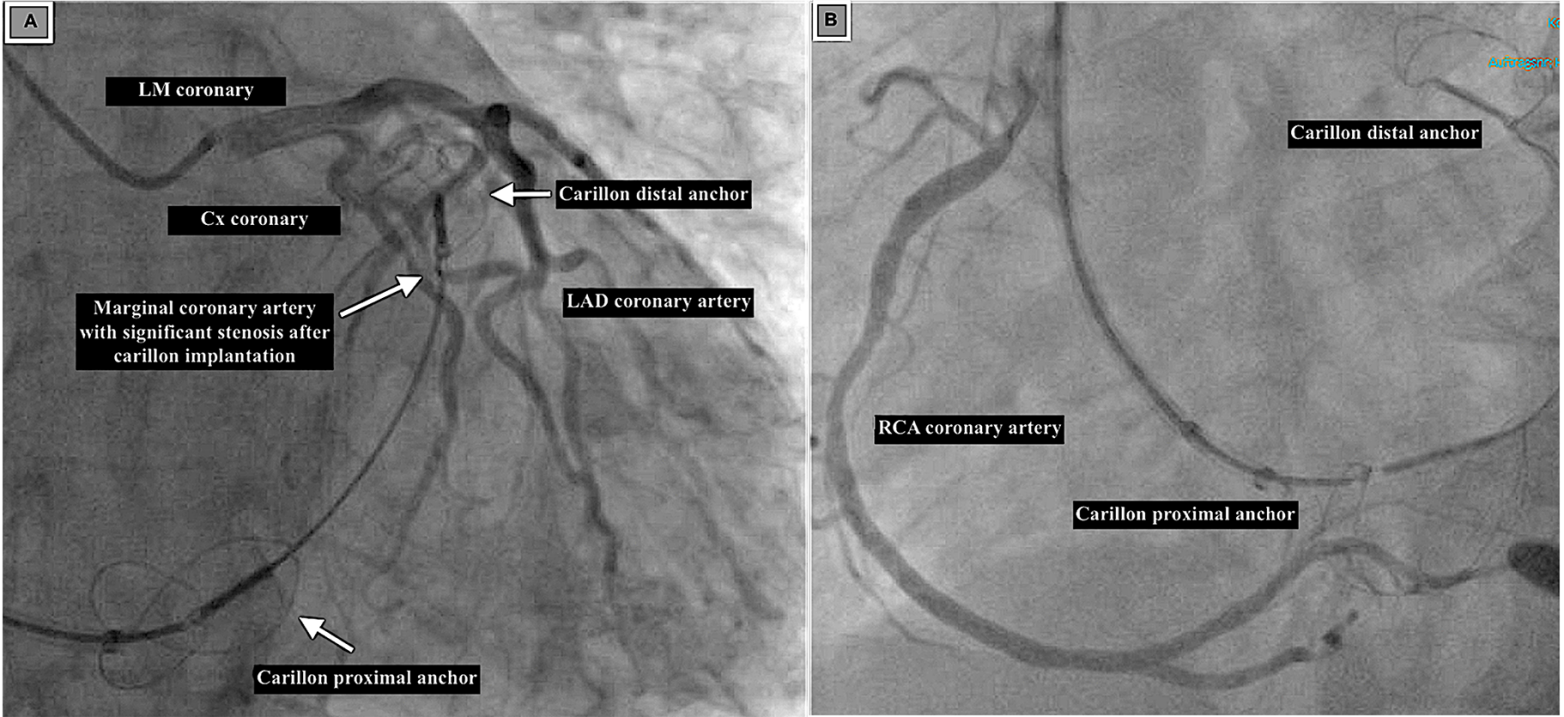
**Coronary arteries in relation to Carillon device implantation**. (A) Coronary angiography showing significant stenosis of the marginal branch of the Cx coronary artery after Carillon device implantation. (B) Coronary angiography showing the RCA course from the Carillon device.

Compression or occlusion of the left circumflex artery during coronary sinus (CS)-based mitral annuloplasty using the Carillon device (Cardiac Dimensions, WA, USA) has also been described in the literature [7]. Extra care should be taken to avoid this iatrogenic complication (Fig. 3A,B).

A retrospective cCT analysis study of 25 patients undergoing Carillon device implantation studied predictors of Cx compromise. It determined the CS-to-Cx distance at the occlusion or compression point or in the distal landing zone in the absence of Cx compromise.

According to CA, 11 patients did not have Cx compromise while 7 patients were identified with Cx occlusion, and 7 had Cx compression. Receiver operating characteristic curve (ROC) analysis identified a CS-to-Cx distance of <8.6 mm, specifically in the distal device landing zone, as predictive of Cx compromise [7].

Clinical anginal pain, ECG changes, and intraoperative CA could help in the diagnosis. Treatment options include repositioning the Carillon device, using another treatment strategy, such as transcatheter edge-to-edge repair, Cx stenting or conservative therapy, if the patient is asymptomatic.

#### 2.2.4 Ischemic Rupture of the Anterolateral Papillary Muscle and Cx

Papillary muscle rupture is a potentially life-threatening mechanical complication following myocardial infarction and is associated with high mortality. It results in severe mitral valve regurgitation, cardiogenic shock and pulmonary edema.

The posteromedial papillary muscle receives blood from the posterior descending artery, whereas the anterolateral papillary muscle receives a dual blood supply from the LAD and Cx arteries. Thus, rupture of the posteromedial papillary muscle is 6–12 times more common. Rupture of the anterolateral papillary muscle is commonly associated with anterolateral myocardial infarction [29].

Vieira *et al*. [30] reported a case of a 59-year-old man who presented with ST-segment elevation myocardial infarction (STEMI) due to obtuse marginal coronary occlusion. Primary coronary angioplasty and stenting were performed. Twelve hours later, the patient developed severe mitral regurgitation due to the rupture of one of the heads of the anterolateral papillary muscle, which was treated with emergency surgery (papillary muscle head reimplantation, mitral annuloplasty with a rigid ring, tricuspid annuloplasty, and coronary artery bypass grafting) [31].

Yamanishi *et al*. [32] reported four cases of severe mitral regurgitation secondary to papillary muscle rupture complicating acute myocardial infarction. The culprit infarction vessel was Cx in 2 patients [33].

Transthoracic echocardiography (TTE) can confirm the diagnosis of papillary muscle rupture with a sensitivity of 65%–85%. However, in some cases, TEE is required to confirm the diagnosis [30]. C-MRI can help identify the underlying pathology.

This could necessitate an emergent surgical or catheter-based intervention. Surgical treatment includes either mitral valve repair or chordal sparing mitral valve replacement. However, mitral valve repair is believed to be superior to mitral valve replacement with respect to improving left ventricular function [32].

#### 2.2.5 Surgical Aortic Valve Replacement in Patients With an Anomalous Cx

An abnormal origin of the Cx from RCA or with retro aortic course is a rare coronary artery anomaly.

In patients with anomalous Cx undergoing surgical aortic valve replacement (SAVR), technical consideration should be given to the use of a smaller prosthesis to avoid compression of the anomalous left circumflex artery and to avoid fatal complications [34]. cCT imaging is mandatory to better demonstrate its origin, course, and spatial orientation compared with the surrounding structure to improve management outcomes.

#### 2.2.6 Left Atrial Appendage Closure (LAA) Devices and Cx

Transcatheter and surgical left atrial appendage closure can jeopardize the Cx artery. A study of 116 cCT scans of patients with left atrial appendage closure devices defined the landing zone plane as parallel to the LAA orifice at the perpendicular course of the Cx’s beginning level.

The data show that landing zones more distal to the orifice of the LAA are safer in terms of Cx damage. Device implantation with a distance of less than 2 mm to Cx was considered dangerous (30.2% of all cases).

Therefore, extra care should be taken during LAA closure to prevent iatrogenic complications [4]. cCT-imaging could help in demonstrating the spatial orientation compared with the surrounding structures to improve management outcomes. Additionally, ECG changes and intraoperative CA could help in the diagnosis. Treatment options include repositioning of the device or use of another device closure mechanism.

**Fig. 4.**
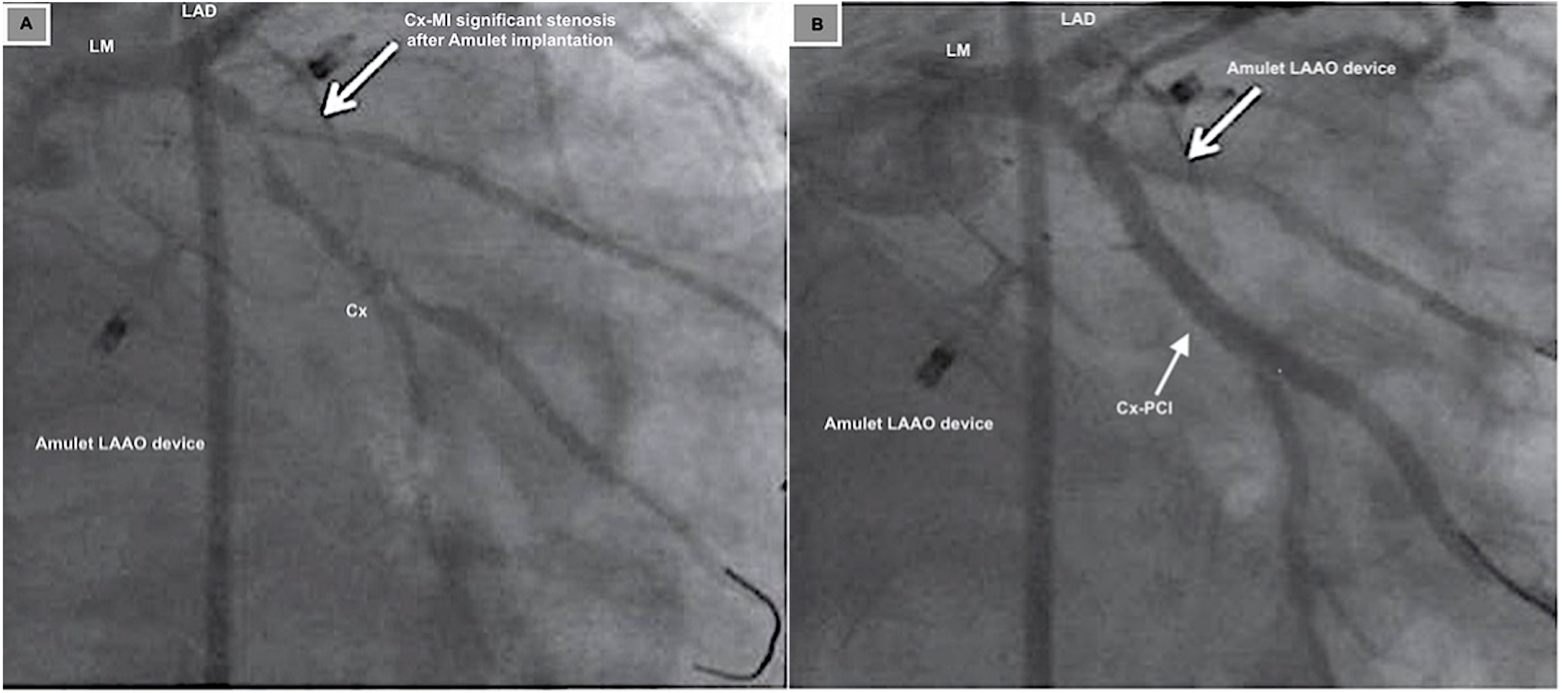
**LAAO using an Amplatzer device in relation to Cx coronary artery**. (A) LAAO using an Amplatzer device was completely deployed. CA confirmed significant Cx-M1 coronary artery stenosis after distal lobe implantation. (B) Cx-PCI [36]. LAAO, left atrial appendage occlusion; LM, left main; LAD, left anterior descending; PCI, percutaneous coronary intervention.

**Fig. 5.**
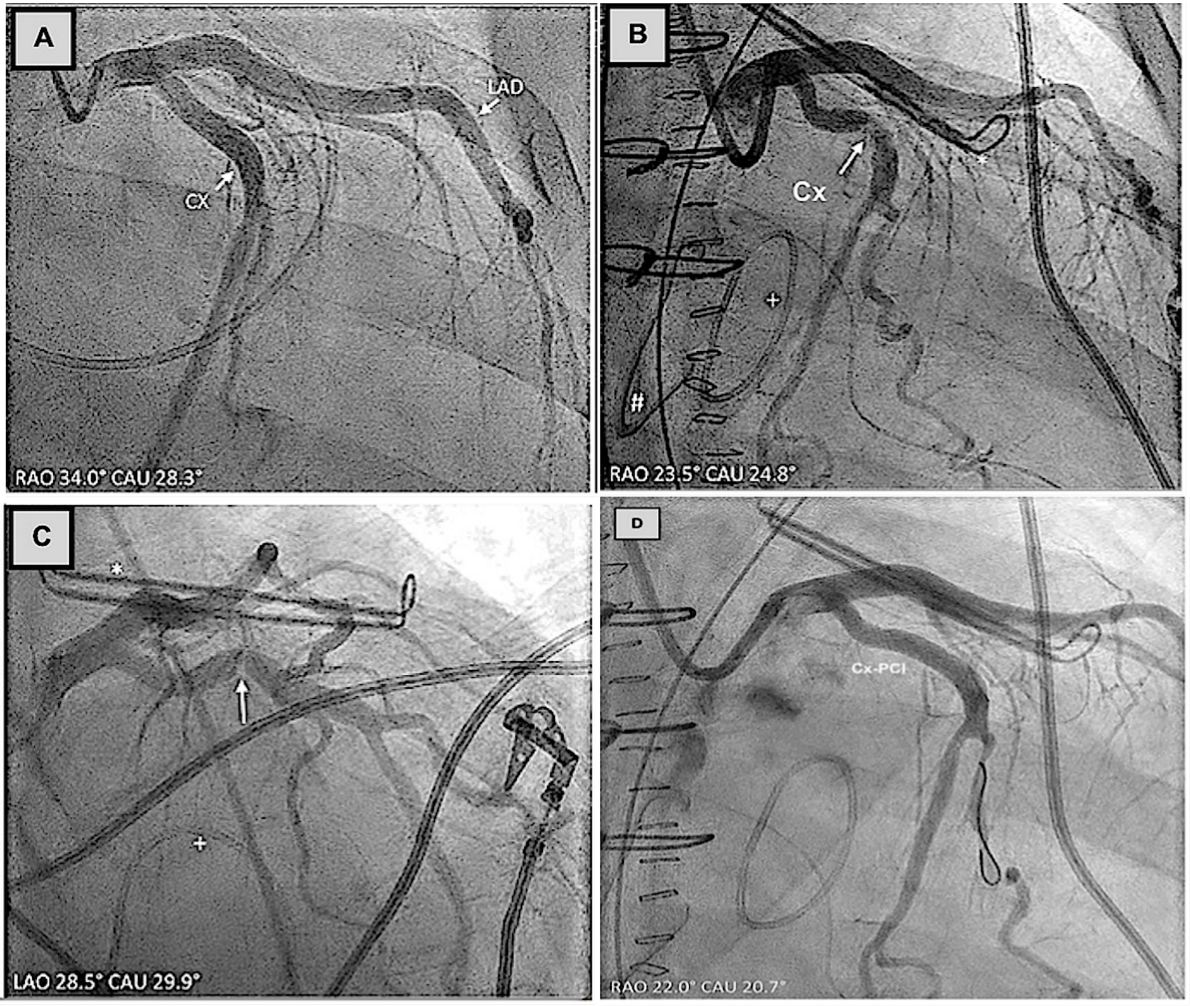
**AtriClip LAAO in relation to left coronary arteries**. (A) Preoperative CA showed non-significant stenosis of the Cx coronary artery. (B,C) CA confirmed significant Cx stenosis after LAAO using the AtriClip device. (D) CA showed good angiographic results after Cx-PCI [35]. LAAO, left atrial appendage occlusion; Cx, circumflex; PCI, percutaneous coronary intervention.

A case report of a 59-year-old man with known non-significant stenosis of the Cx coronary artery who discontinued oral anticoagulant therapy despite atrial fibrillation due to repeated head contusions. LAA closure was planned. During the positioning of the left atrial appendage occlusion (LAAO) of the Amulet device (23 mm) (Abbott, Chicago, IL, USA) (Fig. 4A), ECG changes with inferior wall ST elevations were observed. CA revealed compression of the proximal Cx causing critical stenosis (Fig. 5A, Ref. [35]). After repositioning the device deeper in the ostium, Cx-PCI was performed with no Cx stenosis at the final CA (Fig. 4B) [36].

Kuzmin *et al*. [37] reported a case of delayed Cx artery obstruction after mitral and tricuspid valve surgery along with AtriClip (AtriCure, Mason, OH, USA) implantation. 24 h later, the patient suffered from myocardial infarction. CA confirmed the Cx artery stenosed at the level of the AtriClip device. After Cx-PCI, the final coronary angiogram showed a non-significant Cx stenosis (Fig. 5A–D). Therefore, surgeons should consider placing the AtriClip device slightly far from the base of the left atrial appendage to avoid Cx coronary obstruction [35].

### 2.3 Electrophysiology and Cx 

A study analyzed 5709 consecutive patients who underwent radiofrequency ablation for atrial fibrillation. Heart specimens were dissected to analyze the courses of the coronary arteries. In the pathological specimens with Cx injury of eight patients (0.14%), the distal coronary sinus and anterior left atrium (LA) correlated well with the course of the Cx.

Despite its rarity, this complication could be associated with potentially life-threatening ventricular arrhythmias and acute sinoatrial node (SN) dysfunction requiring permanent pacing. Vigilance and low-power settings are important for minimizing the risk of arterial injury [3].

Spar *et al*. [38] reported a case of a 16-year-old female patient who underwent radiofrequency (RF) ablation in the left lateral area because of Wolff–Parkinson–White syndrome and supraventricular tachycardia. During ablation, the patient developed reversible ST-segment elevation secondary to Cx coronary artery spasm. Coronary angiography revealed that the ablation catheter was in close proximity to the circumflex coronary artery. Subsequently, switching to another technique using cryoablation successfully eliminated conduction via the accessory pathway [36]. Pothineni *et al*. [39] provided a comprehensive systematic review of coronary artery injury related to catheter ablation.

C-MRI could help to determine the ablation strategy, ECG changes, and intraoperative CA during the procedure could help in the diagnosis. Treatment options include intraoperative spasmolytic medication, another ablation strategy, and Cx stenting.

### 2.4 Imaging and Cx 

Imaging plays an important role in the diagnosis and management of Cx. TTE plays a significant role in the initial rapid evaluation and demonstration of the anatomy. Findings include regional wall motion abnormalities, ischemic mitral regurgitation, and hematoma in the Cx region and pericardial effusion in case of GCV injury after Cx-PCI. TEE has higher accuracy and resolution than TTE. CA helps in the evaluation of coronary artery anatomy, the vessel course and identification of the exact site of drainage. However, it may necessitate three-dimensional (3D) reconstruction information, particularly the relationship to adjacent structures.

cCT and cMRI are cross-sectional non-invasive modalities that have high resolutions, multi-planar imaging/reconstruction capabilities, and a wide range of view. ECG gated cCT can be performed rapidly to avoid motion artefacts. The disadvantages of cCT include the use of ionizing radiation and contrast media. The radiation dose can be minimized using several techniques, such as retrospective ECG gating with tube current modulation, prospective ECG triggering, low voltage, automatic tube current modulation, and iterative reconstruction algorithms. In addition, multiplanar reformats and 3D reconstruction techniques enable an exquisite demonstration of the anatomy of the structure [38]. In addition, in particular patients with mitral annular calcification (MAC), CT could help further analyse the anatomical relationships with the Cx, the extent of calcification, quantification of MAC severity and the risk of coronary artery compression and myocardial infiltration, enabling better identification of patient risk for mitral intervention and optimal preprocedural planning, which could lead to improved outcomes [40].

C-MRI requires a gadolinium-based contrast agent. However, cMRI can be obtained without a contrast medium through navigator-gated 3D whole-heart steady-state free precession (SSFP) sequences. In addition, anatomical information can be obtained using bright blood (cine SSFP) or black blood (double inversion recovery) sequences. Moreover, the flow can be quantified using phase-contrast velocity-encoded sequences. Further, stress perfusion imaging can detect myocardial ischemia while infarcts can be identified using delayed enhancement MRI sequences. The disadvantages of cMRI include a long investigation time, occasionally required sedation and artefacts. Dekker *et al*. [41] provided a summary of the measures to reduce the use of gadolinium-based contrast agents without compromising diagnostic quality. Tzimas *et al*. [42] provided a review on three key noninvasive cardiac imaging modalities-cCT, cMRI, and PET/CT-and summarized key publications relevant in clinical practice.

We hope to increase awareness of the clinical implications, interventional challenges, and complexities of the Cx artery compared with other coronary arteries and highlight the importance of imaging information in management strategies during cardiac interventions that could potentially improve patient outcomes.

The main limitation of this review is the lack of sufficient evidence-based clinical practice as most data were derived from single studies, case series and case reports. Therefore, the validation of the data is a matter and further research would strengthen the review’s critical appraisal aspect.

**What is already known on this topic**: The circumflex artery (Cx), in contrast to other coronary arteries, is more vulnerable to injury during radiofrequency ablation, left atrial appendage closure, mitral valve repair, and coronary sinus-based mitral valve intervention. It has a lower success rate in chronic occlusion recanalization.

**What this study adds**: An Overview of Cx artery clinical implications, interventional challenges, and complexities that could influence management strategies in the daily clinical practice. Imaging information about the Cx artery and its relation to the surrounding cardiac structures is useful for a better understanding of the spatial orientation and could help in preventive measures, prediction, prompt recognition, and understanding the injury mechanism if occurred, thus implementing therapy according to the cause.

**How this study might affect research, practice, or policy**: Awareness among interventional cardiologists of Cx artery peculiarities and the importance of imaging information in management strategy could improve outcomes.

## 3. Conclusion

Awareness of the clinical implications and interventional challenges of the Cx artery compared with other coronary arteries is crucial. The artery is more vulnerable to injury during procedures such as radiofrequency ablation, left atrial appendage closure, mitral valve repair, and indirect mitral valve annuloplasty interventions through the coronary sinus. In addition, it has a lower success rate in chronic occlusion recanalization. Imaging information about the Cx artery and its relationship with surrounding cardiac structures is crucial for understanding its spatial orientation. This information plays a significant role in the management strategies during cardiac interventions, potentially improving patient outcomes.

## Abbreviations

AAOCA, An anomalous aortic origin of a coronary artery; CA, Coronary angiography; CTO, Chronic total occlusion; CSA, Coronary sinus angiography; cCT, Cardiac CT; CTA, Computer tomography angiography; CVS, Cardiac venous system; Cx, Circumflex artery; GCV, Great cardiac vein; IVUS, Intravascular ultrasound; LAA, Left atrial appendage; LAD, Left ascending coronary artery; cMRI, Cardiac magnetic resonance imaging; OCT, Optical coherence tomography; PCI, Percutaneous coronary intervention; PDA, Posterior descending artery; PET, Positron emission tomography; PLBs, Posterolateral branches; ROC, Receiver operating characteristic curve; STEMI, ST segment elevation after myocardial infarction; SSFP, Steady state free precession; 3D, Three-dimensional; TTE, Transthoracic echocardiography; LAAO, left atrial appendage occlusion.
